# Supplementary material for: Are carbon emissions trading and green financial instruments synergistic? -Comprehensive quantitative research based on content analysis
Source: PLoS One. 2024 Mar 7;19(3):e0298601. doi: 10.1371/journal.pone.0298601 (PMC10919601; doi:10.1371/journal.pone.0298601)
Supplement: S1 Data — (ZIP) [file pone.0298601.s001.zip › Data/地级市Z_F工作报告.zip!/┴╔─■/░░╔╜╩╨/└┤╫╘╣1⁄2╓┌║┼í╛┬φ┐╦╩2╛▌═°í┐_2019.docx]

各位代表：

现在，我代表鞍山市人民政府向大会报告工作，请予审议，并请市政协委员和其他列席人员提出意见。

一、2018年工作回顾

2018年是全面贯彻党的十九大精神的开局之年。面对改革发展稳定的艰巨繁重任务，我们在省委、省政府和市委的坚强领导下，坚持以习近平新时代中国特色社会主义思想为指导，全面贯彻落实习近平总书记在辽宁考察时和在深入推进东北振兴座谈会上的重要讲话精神，牢牢把握稳中求进工作总基调，按照高质量发展要求，持之以恒落实新发展理念和“四个着力”“三个推进”，保持定力、攻坚克难，有效应对各种风险挑战，新时代鞍山全面振兴迈出了新步伐。

一年来，我们主要做了以下工作。

（一）全力推进经济平稳健康发展

经济运行稳中有进。预计全市地区生产总值增长5.5%，创近四年来最好水平；一般公共预算收入增长8%，创近六年来最好水平；规模以上工业增加值增长10%，创近七年来最好水平；税收收入增长18%，列全省第二位；城乡居民收入分别增长6.9%和7.7%。

工业效益明显提升。规模以上工业企业主营业务收入增长35%，利润增长50%，新增规模以上工业企业93户，工业用电量增长9.7%，工业对经济增长贡献率达到52.1%。鞍钢工业总产值增长18.2%，税收增长30.7%。“双鞍”融合取得积极进展，完成7个项目试生产，德邻陆港一期投入运营。

新动能加快积蓄。市本级投入科技创新经费5145万元，新增院士专家工作站2个、省级研发平台24个、科技成果转化项目88个，新增高新技术企业56家、省级科技型中小企业212家、科技小巨人种子项目落户企业9家，民营规模以上工业企业研发投入增长2.4倍。福鞍集团完成10兆瓦燃气轮机试制，迈格钠磁科技产业园开工建设，数据计算中心综合基地建成运营，德科斯米尔汽车线束项目竣工投产，宏成电力建成世界首条钠离子电池生产线。

现代服务业加快发展。鞍山成为全国首批供应链创新与应用试点市、鞍钢成为试点企业。投资7亿元的万达广场主体封顶，投资2.9亿元的尚嘉购物广场竣工营业，56个服务业重点项目开工建设，鞍山城市花园等一批项目签约落地。全市新增金融机构4家，新设立基金2支，铁东金融集聚区入驻企业20家。七彩化学和亚世光电成为2018年以来东北三省仅有两家A股新上市企业。千山风景区跻身全国百强5A级景区第41位，获评东北地区唯一的“中国天然氧吧”。成功举办“中国首届农民丰收节暨鞍山南果梨丰收节”。千山对桩石村被评为“中国美丽休闲乡村”。全市旅游总收入增长13%。

（二）全力深化改革

重点领域改革取得突破。完成党政群机构改革和事业单位改革，公益性事业单位精简92.1%，新组建鞍勤等15个企业集团，实现事企分开。深化园区体制创新，开发园区数量精简54%。县乡财政管理体制改革深入推进，全市55个乡镇全部实现国库独立运行，一般公共预算收入增长55.6%。

“放管服”改革持续深化。建成全市政务服务“一张网”，在全省率先实现“32证合一”“证照分离”，审批服务办理时限压缩68%，审批环节压缩54.9%，政务服务事项进厅率达到79.8%，“最多跑一次”事项达到93.5%，实现了数据多跑路、群众少跑腿。全面实行“双随机、一公开”监管。企业开办时间压缩至3.5个工作日，新发展市场主体4.4万户，日均增加121户。我市创新小微企业服务工作获国务院通报表扬和国家督查激励政策。

营商环境进一步优化。开展“市长企业家接待日”，实行重点企业领导包保责任制，帮助195户企业解决生产经营难题243个，帮助140户企业获得电力市场交易资格。全面落实减税降费政策，减免税款57.7亿元。组建先进制造创新创业基金。全年新增贷款215.8亿元。全力解决涉企历史遗留问题，投入20.2亿元解决动拆迁项目遗留问题，投入12.5亿元解决已成交未净地问题，投入2.3亿元兑现招商引资政策，投入16.5亿元偿还拖欠工程款，为企业办理不动产登记手续1651件，105件涉党政机关“执行难”积案全部执行完毕，帮助鞍钢、五矿等中省直企业解决占地补偿、矿山征地等突出问题，为企业卸下了历史包袱，营造了宽松发展环境。深入开展“办事难”“特权车”专项整治，出台“八个严禁”工作纪律，处理涉及营商环境投诉158件，办结率达到80%。

国资国企改革不断深化。全面启动市属国有企业“三项制度”、契约化、成本规制等6项改革试点。5家二级企业混改试点进展顺利。“三供一业”分离移交基本完成，接收物业1181万平方米、供热1350万平方米，划转资产23.9亿元。稳步推进53户厂办大集体改革，全面推进“僵尸企业”处置工作。

民营经济不断壮大。成立民营经济发展服务委员会，召开全市加快民营企业发展大会，出台加快民营企业发展的若干政策和措施，隆重表彰6户改革开放40年功勋民营企业和2018年度纳税50强民营企业，全力营造尊重和激励企业家干事创业的社会氛围。建成“企业之家”服务平台，注册企业2万户。建立中小微企业金融信用综合服务平台，实现线上融资16.8亿元。召开银企对接会35次，涉贷金额329.2亿元。新增“个转企”882户，总量全省第三、完成比例全省第一。后英、紫竹两户民营企业进入销售收入超百亿行列，博亿印染入选全国轻工百强企业，10户民营企业纳税超亿元。

（三）全力扩大开放

开放步伐不断加快。主动融入共建“一带一路”，投资7.5亿元的西柳国际物流园及综合物流保税中心项目主体完工，亚马逊、敦煌网等跨境电商平台建成启动，中巴“两国双园”项目加速建设。西柳海关监管场所全面建成，结束了鞍山没有海关监管场所的历史。鞍钢国贸成为我市第一家省级外贸综合服务企业。经开区成为首批辽宁自贸区协同发展重点产业园区。主动融入沈阳经济区建设，组建了物流产业联盟、旅游企业联盟。积极融入“一带五基地”建设，重点推进79个产业基地项目和131个技术攻关项目。深化与南京对口合作，洽谈推进项目30个。

“双招双引”取得进展。成立市招商引资服务中心，组建四大区域招商中心，组织招商活动300余次，签约亿元以上项目147个。对重点项目全部配备项目管家，实施项目投资承诺制和重点项目全程代办，全年开复工亿元以上项目294个。抢抓国家支持东北振兴机遇，向上争取各类资金136.1亿元。全面实施“钢都英才计划”，引进高层次人才157人、国外“高精尖”人才15人，吸引7603名大学生来鞍就业创业。投资3亿元的鞍山人才产业园全面建成，35家研发企业达成入驻意向。

外经外贸势头良好。成功举办“首届中国西柳国际棉服采购节暨东盟加六国特色商品展洽会”，交易额突破7.2亿元。设立“乌兰巴托·西柳服装城”等2个海外分市场。参加首届“进博会”成果丰硕，成交金额排名全省第二。

（四）全力推进乡村振兴

农业现代化深入推进。我市成为全国第二批特色农产品优势区域，海城市成为国家农产品质量安全示范县，台安县成为国家级农村产业融合发展示范园区和农业科技园区，岫岩县经开区成为省级农业科技园区。全市新增设施农业面积5664亩，实施亿元以上农业项目9个，规模以上农产品深加工产业集群主营业务收入增长40.8%。“鞍山南果梨”“岫岩玉”入选中国地理标志产品区域品牌价值百强。

农业农村改革不断深化。我市获批全国农村集体产权制度改革整市推进试点市。初步完成农村集体承包地“三权分置”改革，确权333万亩，流转132万亩。投入4280万元扶持壮大村级集体经济。海城市农村综合改革试点经验获国务院通报表扬，并在全省县域推广。

农村环境逐步改善。新建改造“四好农村路”340公里，改造农村卫生厕所5309户，建成宜居达标村300个。完成人工造林6万亩。农作物秸秆综合利用率达到85%，农村生活垃圾处置体系覆盖率达到70%。清理整治违规建设“大棚房”项目23个。

（五）全力打好“三大攻坚战”

有效防范化解重大风险。建立政府性债务全口径监管机制，多措并举化解政府债务，全市政府性债务风险总体可控。顶着巨大的支出压力，连续三年实行养老金缺口市级统筹，减轻了县（市）区财政负担，保障了退休职工养老金按时足额发放。协调12家银行机构妥善处置企业逾期贷款风险，有效防范企业信贷风险，全市金融环境总体稳定。

脱贫攻坚取得重要成果。市本级投入扶贫资金6158万元，为岫岩县争取少数民族发展资金1938万元。实施产业扶贫4670户，就业扶贫1163人，改造农村D级危房1847户，为2.2万名贫困村民免费健康体检，为6.5万人次贫困人口报销医疗费用3150万元。全市完成9301人脱贫、26个贫困村销号，至此全市81个贫困村全部实现摘帽。我市建档立卡贫困户精准脱贫信息公开工作经验在全省推广。

污染防治工作取得明显成效。全力推进中央环保督察反馈意见整改落实，511个环境信访案件办结率94.9%，治理台安县油泥坑塘12个，处理油泥2.1万吨；649件“回头看”群众信访投诉全部办结。大力推进蓝天保卫战，投入17亿元实施菱镁产业全链条污染治理，投入8.6亿元完成鞍钢炼焦总厂脱硫脱硝等14个治理项目，淘汰10吨以下燃煤锅炉317台，提标改造20吨以上燃煤锅炉140台，全市PM2.5浓度下降12.8%，市区环境空气质量达标率提高到81.9%，同比上升9.8个百分点，创历史最好水平。强力开展重污染河流治理，全面推行河长制，采取河流清淤、排污口封堵等综合治污举措，投入3.2亿元实施运粮河生态环境综合治理，投入6.9亿元实施海城河、五道河21项治污工程，投入近2000万元实施太子河河道及沿岸垃圾整治，关停涉水污染企业66家，全市6个国控断面基本达标，多年来水质恶化趋势得到初步扭转。

（六）全力保障和改善民生

在财政支出压力巨大的情况下，我们尽最大努力保障和改善民生，全年财政用于民生支出比重超过80%。全面开通8890民生问题大数据平台和政务便民服务平台。

人民生活不断改善。全市新增就业4.2万人，城镇登记失业率3.6%。城市低保标准提高4.5%，特困人员供养标准提高5%。帮助500多名退役士兵实现就业，为社区工作者平均提高补贴标准500元，机构供养孤儿基本生活养育标准提高54%。

城市品质不断提升。巩固全国文明城市创建成果，持续推进文明城市建设各项工作。自由东街隧道开工建设，投入3.5亿元建成明山路等19个基础设施项目。改造棚户区6443套、老旧住宅楼70栋、各类管网136公里。重新启动市中心医院门诊病房楼等12个“半拉子工程”。为8883户已售多年的商品房办理了产权证。鞍山机场旅客吞吐量首次突破26万人次，增长31.1%。智慧鞍山建设步伐加快，智慧城市运行管理中心主体建成，城市网格化管理平台投入使用，智慧大交通等项目加速建设。

社会事业全面发展。新建乡镇中心幼儿园9所，改造农村薄弱学校156所，九中新教学楼主体封顶，鞍山师范学院校园改扩建项目进展顺利，职业教育“双元”培育改革试点走在全国前列，职教城承接社会化培训2.6万人次。在全省率先启动中小学课后服务，66人以上“超大班额”全部消除，取缔关停148家校外培训机构，“乱补课”“择校热”“大班额”问题初步解决，一线教师和班主任待遇进一步提高。

深入实施分级诊疗制度，积极推进方便门诊、自助挂号、预约诊疗等医疗服务，取消门诊输液和住院患者押金全额垫付。在全省率先完成达标乡镇卫生院采购医疗设备任务。医联体建设实现城乡全覆盖。城乡居民基本医疗保险政府补助标准提高8.8%。

大力发展医养结合，医疗与养老机构服务协议签约率达到89.9%。加快推进居家养老，规划建设市级居家养老服务指导中心和养老服务大数据平台。全市新增民办养老机构13家，新增养老床位1112张。

建成国内一流标准的鞍山大剧院，引进高水平演艺公司，为百姓提供高端文化演出服务。市群众艺术馆、档案馆、科技馆等项目竣工。奥体中心与中奥集团合作运营，成功举办“一带一路”乒乓球邀请赛、全国乒乓球锦标赛等大型赛事。

社会大局保持稳定。深入开展“扫黑除恶”专项斗争，打掉犯罪团伙40个。扎实推进信访矛盾“减存控增”三年攻坚行动，信访积案化解率提高到55.3%。扎实开展“七五”普法工作，建成市级公共法律服务中心。妥善应对5轮强降雨过程，及时转移群众3.5万人次，实现零死亡目标。有效控制非洲猪瘟疫情。全年未发生重特大安全事故和食品药品安全事件。

广播电视、新闻出版、残联、民族、宗教、外事、侨务、对台、统计、接待、档案、编制、气象、地震、民兵预备役等工作取得新成绩。

（七）全力推进“重强抓”专项行动

坚持把政治建设摆在首位，树牢“四个意识”，坚定“四个自信”，坚决做到“两个维护”，切实履行管党治党政治责任和党风廉政建设主体责任，全面完成中央巡视反馈意见整改落实工作，认真做好中央环保督察、中央信访督察、“扫黑除恶”专项斗争等中央重大专项工作部署。

全面开展“重实干、强执行、抓落实”专项行动，挂图作战、节点考核，有力有序推进重大项目建设、重点改革举措落实，解决了一批难题、办成了一批大事、促进了干部作风持续好转，形成了求真务实、真抓实干、共促振兴的良好局面。

自觉接受市人大及其常委会的法律监督、工作监督和市政协的民主监督，在全省各市率先实行建议提案办理工作第三方评估，570件人大代表建议和政协提案全部按时办结，见面率、答复率、满意率均达到100%。

各位代表，经过全市上下勠力同心、奋勇拼搏，取得了来之不易的成绩，我们要倍加珍惜。这些成绩的取得是习近平新时代中国特色社会主义思想正确指引的结果，是省委、省政府和市委坚强领导的结果，是市人大、市政协有效监督和鼎力支持的结果，是全市人民共同奋斗的结果。在此，我代表市政府向全市广大干部群众，向各民主党派、人民团体和鞍钢等中省直单位，向关心支持鞍山发展的海内外朋友，表示衷心的感谢和崇高的敬意！

在看到成绩的同时，我们更清醒地认识到，面对广大人民群众的新期待，对标高质量发展的新要求，全市经济社会发展仍然存在一些问题、困难和挑战。主要表现在：经济运行持续向好的基础不牢，重大项目储备少、新开工项目少，有效投资不足，全年固定资产投资下降9.8%；新动能不足，传统产业转型升级缓慢；体制机制障碍依然存在，深化改革的任务仍然艰巨；县域经济、民营经济发展不充分，营商环境建设任重道远；民生领域仍有短板，污染防治任务繁重，公共服务供给能力与人民日益增长的美好生活需要还有差距；历史遗留问题仍未彻底解决，各种风险矛盾隐患交织叠加，防范化解难度加大；一些干部思想观念陈旧，作风不实，执行力不强。我们将采取有力措施，全力加以解决。

二、2019年工作安排

2019年是新中国成立70周年，是全面建成小康社会关键之年，做好各项工作意义重大。面对前所未有的发展机遇和风险挑战，我们要坚定信心、下定决心、保持恒心，撸起袖子加油干，补齐拉长“四个短板”，扎实做好“六项重点工作”，以新气象新担当新作为，全力推动鞍山全面振兴、全方位振兴。

今年政府工作的总体思路是：以习近平新时代中国特色社会主义思想为指导，深入学习贯彻习近平总书记在辽宁考察时和在深入推进东北振兴座谈会上的重要讲话精神，全面贯彻落实中央和省、市委经济工作会议精神，统筹推进“五位一体”总体布局，协调推进“四个全面”战略布局，坚持稳中求进工作总基调，坚持新发展理念，坚持推动高质量发展，坚持以供给侧结构性改革为主线，坚持深化市场化改革、扩大高水平开放，深度融入“一带五基地”建设和“五大区域发展战略”，继续打好“三大攻坚战”，以改革为统领，以创新为动力，以项目为支撑，以环境为保障，以民生为根本，深入开展“重实干、强执行、抓落实”专项行动，进一步稳就业、稳金融、稳外贸、稳外资、稳投资、稳预期，提振市场信心，保持经济持续健康发展和社会大局稳定，为全面建成小康社会收官打下决定性基础，以优异成绩迎接中华人民共和国成立70周年。

综合考虑各方面因素，今年全市经济社会发展的主要预期目标是：地区生产总值增长与全省保持同步，一般公共预算收入增长6.5%以上，规模以上工业增加值增长8%，固定资产投资增长10%以上，社会消费品零售总额增长7%，进出口总额占全省的份额进一步提升，城镇登记失业率控制在3.9%以内，城乡居民收入增长与经济增长基本同步。

在新的一年里，重点要做好以下工作。

（一）狠抓招商引资，全力打好项目建设翻身仗

牢固树立“项目兴市、企业富民”理念，坚定不移坚持以项目为核心，深入开展项目攻坚年活动，力争全年开工建设重点项目450个，固定资产投资达到450亿元。

全力发展“飞地经济”。“飞地经济”是优化产业空间布局，推动县域经济发展，调动招商积极性的有效途径。坚持全市“一盘棋”，成立高规格“飞地经济”组织领导机构，设立“飞地经济”专项奖励资金，明确各开发园区产业发展方向，加强基础设施建设，利用闲置的302万平方米土地和30万平方米标准厂房承接“飞地”项目。

深入推进“双招双引”。紧盯重点区域，围绕钢铁、菱镁精深加工等传统优势产业和先进装备制造、新一代信息技术等新兴产业，突出鞍钢等重点企业，发扬“五皮”精神，打出飞地招商、专业招商、以商招商、产业链招商、会展招商、轻资产招商、存量招商“组合拳”，全年谋划招商项目300个以上，签约重点项目200个以上。深入实施“钢都英才计划”，采取“人才+项目+平台+基金”引进模式，带动项目、资金、技术等要素集聚，鞍山人才产业园全年引进高端研发项目50个以上。

抢抓政策红利上项目。围绕国家新一轮支持东北振兴政策举措和5G商用、补齐基础设施短板、生态环保、脱贫攻坚等领域，加强沟通衔接，准确把握政策，从优谋划项目，精心准备项目，全力争取中央预算内投资。

（二）狠抓质量提升，全力推进传统产业转型升级

紧紧扭住发展这个第一要务，加快推进新旧动能接续转换，在“稳”的基础上，实现高质量的“进”。

大力推动制造业高质量发展。以高端化、智能化、服务化、成套化为主攻方向，重点发展冶金矿山成套装备、能源装备、交通运输装备等优势产业，打造国内重要先进装备制造业基地。组建装备制造产业转型升级投资基金，开展装备制造质量品牌提升专项行动，引进高水平设计研发团队，加快推进亚世光电模组、工控车载显示屏、福鞍燃气轮机等重点项目建设。

大力推动钢铁及深加工产业链升级。发挥鞍钢龙头作用，围绕板材、线材、管材和钢结构等4大板块，打造精特钢、涂镀板、热轧型材等10大深加工集群，全力争取成为国家产业金融合作试点，加快建设世界级钢铁及深加工基地，推动宝得高炉项目竣工投产和后英、紫竹高炉项目开工建设。拉长钢铁产业链，大力发展装配式建筑产业，引进杭萧钢构等知名企业，打造国家级新型装配式建筑示范园区；加快推进鞍钢钢绳、帅龙钢绳、发蓝钢带等项目建设。

大力推动菱镁全产业链整合提升。做实做强菱镁集团，提高产业集中度，巩固扩大我市在菱镁行业内的定价权和主导权。规划建设国际镁建材产业园，做精镁质基础原料、做大镁建材，打造世界级菱镁新材料产业基地。推广新型镁砂窑炉示范项目，力争新型窑炉使用率达到40%。组建菱镁产业发展基金，打造菱镁交易平台，发展供应链金融和期货交易。

大力推动精细化工产业发展。依托鞍钢煤焦油、中钢热能和七彩化学精细化工产业园，重点发展煤焦油深加工、氢能综合利用和高端染颜料产业，推进24万吨煤焦油深加工、4万吨针状焦和碳微球等项目投产。加快推进危化企业搬迁改造。

（三）狠抓创新驱动，积极培育发展新动能

深入实施创新驱动发展战略，加快建立产学研一体化创新机制，培育新产业、新业态、新模式，推动经济发展动力变革。

着力提升自主创新能力。加快沈大鞍国家科技成果转移转化示范区建设，积极推进经开区、腾鳌开发区申报省级高新区。大力发展院士经济，积极引进科技领军人物，举办好千山院士论坛活动。深化辽宁科技大学与地方合作，加速推进科技成果转化落地。开展“一企一校”产学研精准对接，推行技术经理人制度，全年新增市级以上创新平台20个、科技成果转化项目80个，新增高新技术企业20家，高新技术产品增加值增长15%。

大力发展文化旅游体育健康产业。把文化旅游体育健康产业作为转型升级、创新发展的主攻方向，推动产业融合联动发展。组建鞍山旅游集团，推进千山旅游集团、温泉集团引进战略投资伙伴，制定《新时代鞍山全域旅游规划》，发挥千山、温泉、钢铁、岫岩玉等旅游资源优势，构建鞍山大旅游新格局，打造中国北方重要旅游休闲目的地城市。建成鞍山城市花园项目，打造城市文化名片。大力发展冰雪经济、夜经济等体验消费。成立旅游行业协会，推进旅游服务标准化。积极开拓国内外旅游市场，打造10个以上旅游产业联盟，实现旅游总收入增长15%以上。

大力推进“双鞍”融合。一如既往全力支持鞍钢改革发展，在人才、项目、市场等方面持续深化地企合作。围绕鞍钢做大产业链配套，加速推进六大共建产业园建设，支持鞍钢重点培育国际贸易、工程、信息化等产业，打造百亿级非钢产业。依托鞍钢集团海工钢国家重点实验室，加快建设军民融合特色产业基地。积极推进地方企业与中省直企业战略合作。

全力培育壮大战略性新兴产业。大力发展新一代信息技术产业，实施“数字鞍山”战略，与360企业安全集团、清华启迪深度合作，加快实施数字科技产业园区等项目，打造“鞍山城市产业云”；依托科大聚龙等企业，着力发展供应链金融、大数据应用与服务。大力发展磁应用产业，加快推进迈格钠磁科技产业园建设，成立磁动力技术研究院，重点发展永磁传动等技术和产品。大力发展新能源电池产业，支持九夷能源、宏成电力等龙头企业发展，打造高端锂电池、钠电池产业集群；积极引进氢能源汽车燃料电池等零配件项目。大力发展节能环保产业，组建市环保集团，以循环产业园为依托，重点发展污水处理成套装备、烟气除尘、秸秆发电设备等环保技术装备，不断提升绿色发展水平。

大力发展现代服务业。推进德邻陆港二期建设，打造“互联网+物流”的现代钢铁物流园区。推进万达广场、华润万象汇、银座大厦等大型商业综合体建成营业，促进实体零售业向智能化大型综合服务体转型。积极与阿里、京东合作，推进大型商超企业实现线上线下融合发展。以西柳千亿元市场建设为重点，大力发展会展经济，推进敦煌网等开展跨境电商业务,推动专业市场转型升级。加快铁东金融集聚区建设，积极引进平安银行等金融机构。大力发展股权投资、融资租赁等新型金融业态。

（四）狠抓改革开放，提升振兴发展活力

牢牢把握改革开放这个关键一招，着力在“巩固、增强、提升、畅通”上下功夫，用市场化改革的办法解决改革中遇到的矛盾和障碍，以高水平开放推动高质量发展。

持续深化“放管服”改革。建立营商环境建设评价机制，推行审批服务标准化。做实县（市）区行政审批局，实行市县关联事项集中联动和集成办理。市场准入领域实施“证照分离”“证照联办”“多证合一”改革，工程建设项目审批时限压减至90个工作日，不动产登记5天办结，100个高频事项实现“最多跑一次”。全市政务服务事项“网上办”“集中办”“一次办”比例达到80%以上，努力实现“办事不出门”“一次不用跑”。继续下放权力，进一步规范涉企执法行为，完善“双随机、一公开”监管机制，禁止随意检查、随意罚款，全力打造“营商环境最优市”。

深化国资国企改革。全面完成市属国有企业内部“三项制度”改革，全面推行契约化管理、预算管理、成本规制等改革，完善法人治理结构，切实增强企业活力。完成5家二级企业混改任务。加快解决国企改革历史遗留问题，完成厂办大集体改革和“僵尸企业”处置。

加快推进民营企业发展。加快民营经济发展改革示范城市建设，发挥民营经济发展委员会作用，组建民营企业专家咨询委员会，落实加快民营企业发展的若干政策和措施，支持现有企业做强做大，促进中小企业加快成长，全年力争“个转企”800户、“小升规”80户，“规升巨”5户。充分利用“企业之家”网站、中小企业网服务热线等载体，畅通民营企业诉求通道。定期开展“市长企业家接待日”活动，点对点解决企业遇到的实际困难。落实政商交往负面清单，全面构建“亲”“清”新型政商关系。加大营商环境整治、曝光、问责力度，坚决整治不作为、乱作为、慢作为问题，严厉查处破坏营商环境的人和事。

全面加强开放合作。学习借鉴自贸区改革成功经验，搭建投资贸易便利化综合服务平台。深耕日韩俄、狠抓中东欧，主动参与辽宁“一带一路”综合试验区建设，组建市外贸集团，全力争取获批国家市场采购贸易试点，启动大连海关（西柳）监管区，加快建设中东欧16国进口商品展示馆和东北进口商品展示中心。主动融入沈阳经济区，加快建设中德产业园鞍山分园、远洋智能大型工业焊接机器人研制与产业化等重点项目。重启与国内外友好城市合作交往，深化与南京对口合作，加速发展健康产业等新业态。

（五）狠抓乡村振兴，着力壮大县域经济

大力发展现代农业。落实国家粮食政策，确保全市粮食播种面积310万亩、总产量120万吨。大力推进农业现代化，加强农田水利基础设施建设，新建设施农业5000亩。大力推进农业标准化生产，争取岫岩软枣猕猴桃、台安绿茄等成为国家地理标识产品，做大做强南果梨、观赏鱼、宠物等特色品牌。

推进农村一二三产业融合发展。以腾鳌、桑林、大洋河3个乡村振兴战略示范乡镇建设为牵动，加快推进安井集团5万吨速冻食品、九股河食品3万吨禽肉熟食制品、辽宁天龙中草药种植深加工产业园等项目建设，着力打造东部山区生态农业基地、中西部平原高效农业基地、城市近郊都市农业基地。深入开展省级休闲农业与乡村旅游星级示范创建工作，加快建设君子兰山庄等特色小镇。

深化农村改革。做好国家整市推进农村集体产权制度改革试点，完成新型集体经济组织等6项重大改革任务。推广海城市农村综合改革经验，推进土地规范流转、增值流转，开展农业设施权属认证、融资等工作。继续深化供销社综合改革。

深入实施美丽乡村建设。持续推进农村人居环境整治，新建宜居达标村300个，建成美丽乡村70个。新建“四好农村路”100公里。进一步推进“厕所革命”，重点解决好农村中小学校厕所问题。

（六）狠抓“三大攻坚战”，着力打好重点战役

坚决打好防范化解重大风险攻坚战。全面落实政府债务化解三年行动计划，严控增量、消化存量，依法处置隐性债务，切实防范化解政府债务风险。继续实行养老金缺口市级统筹，通过加强财政保障、养老保险费征缴、严格基金支出管理等方式，千方百计筹措资金，确保企业养老金按时足额发放。坚决打击企业恶意逃废债行为，建立金融风险预警大数据平台，设立上市企业纾困基金，坚决守住不发生系统性、区域性金融风险的底线。

坚决打好精准脱贫攻坚战。聚焦“两不愁三保障”，加大市本级投入力度和少数民族地区扶贫力度，突出抓好产业扶贫、就业扶贫、健康扶贫，重点解决贫困户子女上学、就业和大病用药吃药等问题，防止因病致贫、因病返贫，确保未脱贫的2000多名建档立卡贫困人口全部脱贫、岫岩县省级扶贫开发工作重点县摘帽。进一步完善稳定脱贫政策，开展脱贫攻坚“回头看”，确保全市已脱贫的2.5万人稳定脱贫。

坚决打好污染防治攻坚战。以更大的力度做好中央环保督察整改落实工作，确保全面高质量完成整改任务。加大环保执法力度，坚决遏制超标排放污染物等违法行为。巩固蓝天保卫战成果，完成19个大气污染治理项目，加快淘汰建成区20吨及以下燃煤锅炉，不断提升城市空气质量。打好辽河流域综合治理攻坚战，推进“五河同治”“河岸同治”，实施12个水污染治理项目，推进11座污水处理设施建设和提标改造，全面改善河流水质。持续推进青山工程，着力提高森林城市建设水平，完成造林4万亩，森林抚育1.2万亩；开展“五矿同治”，实施非煤矿山综合治理，引进投资10亿元的青岛冠中100万平方米菱镁矿山生态修复项目。深入推进净土保卫战，全面完成台安纳污坑塘整治。

（七）狠抓民生改善，着力解决民生领域重点问题

把保障和改善民生作为政府一切工作的出发点和落脚点，充分发挥8890民生问题大数据平台和政务便民服务平台作用，推进政务便民服务升级，不断提升人民群众的获得感、幸福感、安全感。

不断提高就业和社会保障质量。严格落实退役军人安置政策和军人优抚优待政策，统筹做好就业困难人员、失业人员、被征地农民就业促进工作，新增城镇就业3.9万人。强化民生兜底保障，继续提高城乡低保、社会救助、退休人员基本养老金等标准。建立农村留守儿童、困境儿童、孤儿关爱服务体系，提高散居孤儿供养标准。

优先发展教育事业。推进学前教育公益普惠制发展，新建10所公办幼儿园。持续整治“乱补课”“择校热”“大班额”等问题，56人以上“大班额”全部消除。建成市特殊教育学校，填补我市高中阶段特殊教育空白。规划建设二十四中和九中艺体馆，完成九中教学楼、三中体育馆建设，支持鞍山师范学院校区改扩建。努力提升职业教育办学层次和人才培训质量。加强师德师风建设，推进廉洁文化进校园，强化全员全方位全过程师德养成。

深入推进“健康鞍山”建设。开展省级健康城市建设试点。全面深化医药卫生体制改革，推进公立医院综合改革，强化重点专科建设。加强基层卫生服务体系建设，构建分级诊疗新格局，加快推进22所乡镇卫生院和社区卫生服务中心标准化建设，着力解决“看病难”问题。完善基本药物制度，健全药品供应保障机制，降低大病慢性病医疗费用，着力解决“看病贵”问题。加快发展智慧医疗，提高远程影像诊断服务水平；强化医德医风建设和行业自律，为百姓提供优质的卫生与健康服务，着力解决“看不好病”问题。

不断丰富人民群众文化生活。开展“文化惠民季”系列活动，建设70个村级文化广场，把鞍山大剧院打造成高雅艺术的演出中心，把群众艺术馆打造成钢都百姓的大舞台。把奥体中心打造成国内外大型赛事的承办地。举办鞍山市第十一届运动会。新增改造市民健身器材6000件。加快国家级乒乓球基地建设，打造“国球名城”。推广校园足球，建立足球青训体系，建设全国五人制足球训练基地。

加快完善养老服务体系。构建以居家为基础、社区为依托、机构为补充、医养相结合的养老服务体系。建立市级居家和社区养老服务指导中心及智慧服务平台，建设县区服务中心5个、社区服务网点50个。打造医养结合特色医院。全力争取进入国家居家和社区养老服务改革试点城市。

继续提升城市品质。深入推进“多规合一”，启动新一轮城市总体规划修编。加快推进自由东街隧道、海岫铁路建成通车，开工建设鞍山机场改扩建、解放路立交桥大修等重点项目。改造棚户区1350套，改造农村危房1727户，为1万户已售商品房办理产权证。对6万户居民室内供水设施进行大修改造，解决高层住宅“用水难”问题。加快智慧城市建设，成立智慧城市运行管理中心，建成智慧大交通项目并投入使用。提高城市精细化管理水平和城市文明程度，着力解决私搭乱建、停车难等问题。

切实维护社会安全稳定。加强“平安鞍山”建设，深入开展“扫黑除恶”专项斗争，深挖涉黑涉恶腐败问题和背后“保护伞”。积极化解信访积案，积案化解率提高到75%。健全基层社会治理体系，开展社区居民委员会和村民委员会换届。深入开展“七五”普法工作。强化安全生产，创建全国食品安全城市，维护社会大局和谐稳定。

（八）狠抓“重强抓”专项行动，着力建设人民满意政府

坚持旗帜鲜明讲政治，以“重实干、强执行、抓落实”专项行动为抓手，不断加强法治政府、诚信政府、廉洁政府建设，推动政府工作提速、提质、提效。

坚定不移把政治建设放在首位。树牢“四个意识”，坚定“四个自信”，坚决做到“两个维护”，始终在政治立场、政治方向、政治原则、政治道路上同以习近平同志为核心的党中央保持高度一致。严格履行全面从严治党主体责任，持之以恒落实中央八项规定精神和省委实施细则。切实履行“一岗双责”，深入推进政府系统党风廉政建设，持续净化修复政治生态。

加快建设法治诚信政府。坚持依法行政，依法接受市人大及其常委会法律监督、工作监督，自觉接受市政协民主监督，主动接受群众监督和舆论监督，确保人大代表建议和政协委员提案办理满意率达到100%。坚持言必信、行必果，政府作出的承诺坚决兑现。坚持“新官理旧账”，拿出最大诚意，做出最大努力，争取最短时间、最大程度解决好招商引资政策未兑现、土地批而未供、拖欠工程款等历史遗留问题。坚持守信激励和失信惩戒，打造诚实守信的社会环境，积极创建国家社会信用体系建设示范城市。

坚持不懈转变工作作风。大力弘扬孟泰精神，凡事讲认真、凡事讲质量、凡事讲效能。大力弘扬创新精神，进一步解放思想、更新观念、拓宽视野，求新求进。大力弘扬实干精神，叫响“实干兴鞍”，做到说一件、做一件，干一件、成一件，以实干谋发展、以实干促振兴。

各位代表！让我们更加紧密地团结在以习近平同志为核心的党中央周围，高举习近平新时代中国特色社会主义思想伟大旗帜，在省委、省政府和市委的正确领导下，解放思想、转变观念，振奋精神、真抓实干，展现新形象、干出新业绩，重塑鞍山环境、重振钢都雄风，为加快推进新时代鞍山全面振兴、全方位振兴而努力奋斗，以优异成绩向中华人民共和国70周年华诞献礼！
